# Risk and Protective Factors for Burnout Among Parents of Children With Complex Care Needs: Parents' Perspectives

**DOI:** 10.1111/cch.70143

**Published:** 2025-07-24

**Authors:** Nathalie J. S. Patty, Karen M. van Meeteren, Minke Verdonk, Marjolijn Ketelaar, Carlo Schuengel, Agnes M. Willemen

**Affiliations:** ^1^ Faculty of Behavioural and Movement Sciences, Section Clinical and Family Studies Vrije Universiteit Amsterdam Amsterdam the Netherlands; ^2^ Amsterdam Institute of Public Health Amsterdam the Netherlands; ^3^ Expert by experience Parent of a child with complex care needs the Netherlands; ^4^ UMC Utrecht Brain Center University Medical Center Utrecht Utrecht the Netherlands; ^5^ De Hoogstraat Rehabilitation Center of Excellence for Rehabilitation Medicine Utrecht the Netherlands

**Keywords:** burnout, caregiver burnout, parental burnout, parenting, parents of children with complex care needs

## Abstract

**Background:**

Parental burnout has been proposed as resulting from a persistent imbalance between stress‐enhancing factors (demands/risk factors) and stress‐alleviating factors (resources/protective factors). Parents of children with complex care needs (CCN) face this imbalance more often than parents in general. To address this, we need to know which factors are considered risky and protective for burnout from the perspectives of parents of children with CCN. To facilitate targeted interventions, this study sought to explore both risk and protective factors associated with burnout as perceived by parents of children with CCN.

**Methods:**

We conducted semi‐structured interviews with 38 parents, who recognized or identified themselves with burnout‐related thoughts and feelings. Parents were selected for maximal variation, based on parental, child and family characteristics. The data was analysed through inductive thematic analysis.

**Results:**

Fifteen themes, divided into three categories, were identified: (1) ‘the parent’ encompassing factors intrinsic to the parent, such as emotional factors and internal drivers, and physical health; (2) ‘the environment interacting with the parent’ including organization of care, social support and socio‐economic factors; and (3) ‘the sum of all factors’—themes underscoring the collective impact of the context including the caregiving and parental responsibility, and the perception of no option but to endure. Most factors were identified as both risk and protective factors, underscoring the dynamic nature of burnout.

**Conclusion:**

Participating parents ascribed burnout to unique combinations of risk and protective factors. Notably, these factors extended beyond the personal sphere to encompass societal structures that current conceptual models for dealing with burnout often ignore. From the perspective of parents, broad multisystem approaches to address parental burnout would likely appear most relevant.

## Summary

- Burnout among parents of children with CCN stems from a complex interplay and accumulation of several risk factors and depletion of protective factors, which vary per parent.
- While certain factors are parallel to those observed in parents of children without CCN, others are distinctly tied to the unique demands of parenting and caregiving a child with CCN, such as the organization of health and social care and the nature and severity of the disability/disease of the child.
- Risk and protective factors expand beyond factors inherent to the parent, involving wider societal structures. This implies that prevention and recovery also need to be promoted at societal and organizational levels, not only at the individual family level.
- Given the broad range of factors that parents themselves consider important, practitioners are encouraged to adopt a broad and open perspective when discussing possible causes of burnout.

## 1 Introduction

Children with complex care needs (CCN) have long‐term physical, developmental, behavioural or emotional problems and require (health)care services beyond what is typical (McPherson et al. 1998). These needs pose a wide range of challenges for parents. These challenges may include having to perform extensive daily assistance (such as helping an adult child to the toilet) and medical complex care tasks while being uncertain about the course of illness or disability (e.g., Hodapp et al. 2019; Power et al. 2019), on top of the normative tasks of parenting. Consequently, parents often find themselves with limited time to tend to their own needs and to those of other family members (Woodgate et al. 2015). Parents of children with CCN have reported elevated levels of parenting stress compared with parents without children with CCN (Cousino and Hazen 2013), which has, in turn, been found associated with parental burnout (Desimpelaere et al. 2023). A review by Patty, van Meeteren, Willemen, et al. (2024) found that 20%–77% of parents of children with CCN experience burnout. Parental burnout has been linked to consequences such as suicidal thoughts, substance abuse, domestic conflicts, and violence and neglect toward the child (Mikolajczak, Brianda, et al. 2018). Studies of risk, alleviating and protective factors for burnout have mostly focused on specific diagnostic and demographic groups (Mroskova et al. 2020), quantitatively assessing predefined factors (see Patty, van Meeteren, Willemen, et al. 2024). To address the challenges experienced by parents, it is important to know what they perceive as factors that may influence burnout. This study seeks to address this gap by investigating the perceived risk and protective factors for burnout among parents of children with CCN.

Parental burnout has been described as the outcome of long‐term exposure to stress, involving emotional exhaustion (EE) in the parental role, emotional distancing (ED) from the child and lack of personal accomplishment (PA) within the parental role (Roskam et al. 2017). Among parents of children with CCN, specific caregiving and parenting aspects of the child play a role, such as the long‐term responsibility of the child which parents cannot relinquish and are difficult to share (Abdoli et al. 2020; Patty, van Meeteren, Verdonk, et al. 2024). Moreover, burnout has been characterized by parents of children with CCN as having a long‐term reoccurring nature, commencing with symptoms of stress that evolve into exhaustion and culminating in a survival mode for some parents. In the survival mode, parents strive to maintain an appearance of control and well‐being while distancing themselves physically and emotionally from themselves and others, including their children (Patty, van Meeteren, Verdonk, et al. 2024).

Among parents in general, parental burnout has been ascribed to a persistent imbalance, wherein stress‐enhancing factors (risk factors/demands) surpass stress‐alleviating factors (protective factors/resources) (Mikolajczak and Roskam 2018). Factors found associated with burnout include mainly *personality characteristics* such as personality traits of parents (e.g., emotional intelligence, neuroticism) (Le Vigouroux et al. 2017; Le Vigouroux and Scola 2018; Mikolajczak, Raes, et al. 2018), perfectionism (Hubert and Aujoulat 2018; Sorkkila and Aunola 2020) and reappraisal (Lin et al. 2022). Moreover, factors pertaining to *parenting and child‐rearing practices*, such as parental cognitions (e.g., self‐efficacy beliefs and positive parenting) have also been linked to burnout (Mikolajczak, Raes, et al. 2018). External factors concerning the *family and social network*, encompassing child and partner relationships, including family functioning, marital (dis)satisfaction and co‐parenting (dis)agreements (Mikolajczak, Raes, et al. 2018), as well as social support (Lin et al. 2022) and the child's personality (Le Vigouroux and Scola 2018), have also been linked to burnout. Socio‐demographic factors, such as being a parent of young children (< 5 years) (Mikolajczak, Raes, et al. 2018), working part‐time/being unemployed (Lebert‐Charron et al. 2018; Mikolajczak, Raes, et al. 2018) and experiencing financial strain, have been shown to be associated with parental burnout (Sorkkila and Aunola 2020).

In studies thus far, risk and protective factors among parents of children with CCN resemble those found among parents in general. These include *personality characteristics* (Findling et al. 2023; Gérain and Zech 2018; Lindström et al. 2011), external factors concerning *family and social network* (Findling et al. 2023; Gérain and Zech 2018; Lindström et al. 2011) and *socio‐demographic* factors, such as having financial concerns (Lindström et al. 2011). Unique factors have been found as well in the form of sleep disruption due to caring responsibilities for the child with CCN (Lindström et al. 2011), the presence of comorbidity and having several children with CCN (Gérain and Zech 2018), and challenges in social participation and limited leisure time (Findling et al. 2023; Lindström et al. 2011). Specific protective factors were mental health support (Yamoah and Brown 2022) and maintaining a positive mindset (Findling et al. 2023). Moreover, perceived consequences of the child's CCN, rather than objective features of children's conditions, have been linked to parental burnout (Gérain and Zech 2018; Mroskova et al. 2020).

Studies on risk and protective factors have been limited to specific diagnostic and demographic groups of parents of children with CCN and focused on parent characteristics more than on broader societal factors (Patty, van Meeteren, Willemen, et al. 2024). While quantitative studies are necessarily selective in the number of factors that can be studied, research into the risk and protective factors or mechanisms that parents perceive as relevant may help to identify blind spots. To address the broad variability within this population, perspectives of parents with and without burnout symptoms need to be included. Next to validating well‐known factors, exploring factors with parents may also reveal novel targets for proactive interventions and policies to reduce and prevent burnout. Hence, the objective of this study was to gain a comprehensive understanding of risk and protective factors contributing to burnout, as perceived by parents of children with CCN.

## 2 Methods

### 2.1 Study Procedures

This study is part of a larger qualitative study on burnout among parents of children with CCN, investigating also how burnout is conceptualized according to parents of children with CCN. Ethical approval was obtained from the Scientific and Ethical Review Board of the Faculty of Behavioural and Movement Sciences of the Vrije Universiteit Amsterdam (registration numbers: VCWE‐2021‐078 and VCWE‐2022‐015).

Parents were recruited through several parental organizations, social media and schools for children with CCN. Parents were eligible to participate if they had a child with CCN and if they recognized or identified themselves with burnout‐related symptoms based on their own perceptions and assumptions, irrespective of having or not having experienced burnout. Both parents who had and had not experienced burnout were included. For parents who had encountered burnout, our interest lay specifically in comprehending the factors they believed contributed to the burnout, as well as what helped them recover. For parents who had not experienced burnout, we focused on the attributes they believed shielded them from burnout. Furthermore, to prevent interference of participation with recovery from burnout and professional help, participants were excluded if they had received any psychiatric treatment in the past 6 months related to burnout, were on a waitlist for such treatment or evidenced suicidal ideations.

We employed purposive sampling to select a varied group of parents based on factors such as parental gender, ethnicity, child age, type of CCN, sibling status and living arrangements. Once the data collection started, a snowball sampling method was applied to identify more potential participants. After including 15 parents, additional participants were included until the analysis of three additional interviews did not generate new content or insights, such as codes or nuances of the meaning of the codes (Hennink et al. 2017). Saturation was determined jointly by A, B and C [blinded for peer review] who participated in each step of data analysis and were familiar with the data. A more detailed prescription of the methods used in this study is provided in the Open Science Framework (OSF) protocol and can be accessed via: https://osf.io/xd8t5/?view_only=21ce436f383b40f0904f9910678b8578.

### 2.2 Instruments

Two partially overlapping topic lists were created by the research team, which included experts in experience and healthcare professionals, and pilot‐tested with a parent of a child with CCN. One topic list was for those participants who identified with having experienced burnout and another for those who recognized burnout symptoms but did not identify themselves as having experienced burnout (see Data S1). Video interviews were conducted through Microsoft Teams or Zoom between May 2021 and June 2023. Each interview involved two interviewers with varying compositions consisting of students, a researcher, experience‐expert parents or a healthcare professional working with parents of children with CCN. All interviewers were trained by A.

Each participant was asked to complete the Parental Burnout Inventory^1^ online (PBI: Roskam et al. 2017). The PBI was used to assess the variation in burnout scores. The PBI has been validated in the Netherlands among parents of children without CCN (Van Bakel et al. 2018). It comprises 22 questions covering three dimensions: (1) EE (eight items, e.g., ‘I feel emotionally drained by my parental role’), (2) ED (eight items, e.g., ‘I do not really listen to what my children tell me’) and (3) PA (six items, e.g., ‘I look after my children's problems very effectively’). Burnout is reflected by high scores on EE and ED and low scores on PA. Response categories range from 0 (*never*) to 6 (*every day*). Internal consistency in the present study was *α* = 0.90 for the whole PBI. The PBI scores can be found in Table 1.

**TABLE 1:** Demographic characteristics and PBI scores of participants (*N* = 38).

	*n* (%)	M (SD)	Min–max
Parent			
Mothers	30 (79)		
Age at interview in years		44.34 (8.29)	30–74
Ethnicity western^a^	34 (90)		
Employed (paid)	31 (82)		
Family			
Married/cohabiting	33 (87)		
1 child with CCN	32 (84)		
≥ 2 children with CCN	6 (16)		
Child with CCN (partially) living at home with parent^b^	31 (84)		
Child			
Age of child with CCN in years		12.66 (10.23)	1–47
Motor or sensory disability/impairment^c^	6 (14)		
Chronic or severe illness	8 (18)		
Intellectual and developmental disorders	21 (48)		
Comorbid conditions	9 (20)		
PBI scores^d^			
Total score (possible range 0–128)		38.39 (19.41)	0–85
Emotional exhaustion (0–46)		20.53 (13.29)	0–46
Emotional distancing (0–46)		10.32 (6.37)	0–31
Personal accomplishment (0–36)		7.55 (5.02)	0–20

^a^ Western encompasses participants descending from North America, Oceania, Indonesia, Japan or Europe (excluding Turkey) (CBS 2024).

^b^ Based on *n* = 37.

^c^ The descriptions of parents were used to indicate whether the disability was primarily motoric, somatic or developmental and/or characterized by comorbid conditions.

^d^ Higher scores indicate more burnout symptoms; a lower score indicates less burnout symptoms.

### 2.3 Qualitative Analysis

The semi‐structured interviews were transcribed verbatim, pseudonymized and then analysed using the six phases of inductive thematic analysis (Braun and Clarke 2021). These phases were conducted in different compositions of the project group (see Section 2.4) to ensure different perspectives and prevent early convergence. First, A and two Master students read and familiarized themselves with the transcripts, making initial notes of possible codes and themes. Then one transcript was open coded for pilot testing. The transcript was then discussed by A, B, C, the Master students and a healthcare professional from the project group to understand alternative coding and interpretations of the text fragments, to encourage thoroughness in the coding process and to establish general rules for open coding. Following this exercise, all interviews were coded independently by A and a Master student using ATLAS.ti 23 for Mac (Scientific Software Development GmbH 2023). Second, when approximately 70% of the transcripts had been open coded, A randomly drew two transcripts to create subthemes. The subthemes were collaboratively created by combining open codes into categories. After all the transcripts had been open coded, A read through all the open coded transcripts, checked the initial open codes, and added or adjusted the open code when necessary, and then categorized all the open codes into the agreed subthemes. New subthemes were created when needed. Third, once all the transcripts had been coded according to subthemes, A, B and C used the list of subthemes to generate initial themes in Excel. Thereafter, and fourth, the initial themes were further refined, and potential connections between themes were discussed with the entire project team. Finally, respondents received a draft of the results and were encouraged to reflect on whether their perspectives and experiences were accurately represented in the draft. The feedback from the respondents was assessed and the results were adjusted accordingly.

### 2.4 Reflexivity

The project team consisted of eight people with expertise in psychology, special education, quantitative and qualitative research, intellectual and physical disability, rehabilitation and nursing. Two of the project members were experience‐expert parents (B, D), two were healthcare professionals working with families of children with CCN, and four were researchers with varying levels of experience in research in the field of families of children with CCN (A, C, E, F). Five students (three Master students and two Bachelor students) with diverse educational and ethnic backgrounds participated in data collection. A and the students involved in data collection and analysis had limited prior experience with research in parents of children with CCN compared with other project members. Continuous reflective discussions occurred throughout all research phases.

## 3 Results

### 3.1 Participant Characteristics

Seventy‐one parents expressed their interest in participating. One was ineligible, eight did not respond to repeated contact attempts, and 24 parents were not included because data saturation was achieved. Thirty‐eight parents participated in the study. There was large variability in the children's diagnoses. See Table 1 for participant and child characteristics.

### 3.2 Key Themes

**FIGURE 1:**
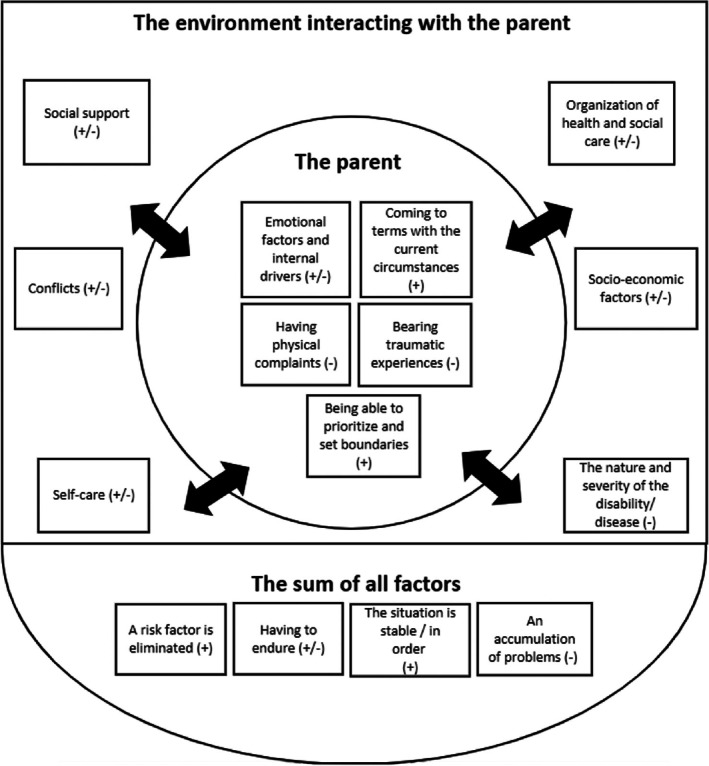
An overview of risk and protective factors.

We identified 15 themes consisting of both risk and protective factors for burnout, or both. The themes have been divided into three main categories: ‘the parent’ encompassing themes inherent to the parent; ‘the environment interacting with the parent’, representing the context of the parents' outer world encompassing external factors, influences and surroundings; ‘the sum of all factors’—themes underscoring the collective impact of the context. The first two categories highlight the various individual factors. Themes were interrelated not only within categories but also across categories. Figure 1 illustrates the complex interplay of all themes. An overview of the categories, themes and the underlying codes reflecting the coding process of parents' perceptions on risk and protective factors for burnout can be found in Table 2. The results section has been divided according to the three main categories, along with illustrative quotes identified by the notation ‘P’ for the parent and ‘#’ for the corresponding number.

**TABLE 2:** An overview of the categories, themes and codes.

Categories	Themes	Codes
The environment interacting with the parent	Organization of health and social care	Poor organization of health and social care^b^ The complex laws, regulations and bureaucracy^b^
		(Health)care is well organized^a^
	Social support	Receiving help and asking for help^a^
		Receiving support and understanding from the formal and informal environment^a^
		A lack of understanding and insufficient support from the formal and informal environment^b^
	Socio‐economic factors	Having financial concerns^b^
		Being able to make choices in the balance between work and personal life^a^
	Conflicts	Not having conflicts^a^
		Having conflicts^b^
	The nature and severity of the disability/disease	The nature and severity of the disability/disease^b^
	Self‐care	Having [mental] space to take good care of yourself^a^
		Not able to take [mental] space for yourself^b^
		Having multiple care tasks and concerns^b^
The parent	Emotional factors and internal drivers	Having a positive outlook on life^a^
		Recognizing and being open about what you feel in your mind and body^a^
		Experiencing guilt and feelings of shame^b^
		Setting high demands/being perfectionistic^b^
		Having a disturbed emotion regulation^b^
		Feeling insecure^b^
		Experiencing chronic sorrow^b^
	Being able to prioritize and set boundaries	Being able to prioritize and set boundaries^a^
	Coming to terms with the current circumstances	Coming to terms with the current circumstances/choosing to let go of resistance or struggle against the situation^a^
	Bearing traumatic experiences	Bearing traumatic experiences^b^
	Having physical complaints	Having physical complaints^b^
The sum of all factors	An accumulation of problems	An accumulation of problems^b^
	Having to endure	Having to endure^c^
	A risk factor is eliminated	A risk factor is eliminated^a^
	The situation is stable/in order	Create a balanced life/balancing between capacities and challenges (i.e., balance between what a person can handle and the challenges they are facing)^a^

^a^ Considered a protective factor.

^b^ Considered a risk factor.

^c^ Considered simultaneously a risk and protective factor.

#### 3.2.1 The Parent

Five themes inherent to the parent were identified, as delineated below.

**Emotional factors and internal drivers.** Parents mentioned having difficulties with emotion regulation, such as difficulty feeling and expressing emotions, being sensitive to stimuli or lacking the time to process emotions. Some parents also mentioned having the desire for complete control over the situation and insisting and believing that everything had to be handled and done independently by themselves. Furthermore, some parents highlighted feeling insecure in their role as a parent, caregiver and a decision‐maker for their child. Parents also mentioned that the comprehensive care demands of the child made them have limited time for their other children, causing feelings of guilt and shame. Moreover, a subset of parents mentioned that they regularly experienced chronic sorrow, entailing recurring feelings of grief and loss, for example, when regular milestones were not achieved by the child with CCN. These were perceived as risk factors for burnout.

Protective factors within the theme involved learning to cope with one's emotions, recognizing and openly expressing emotions, trusting one's intuition, recognizing burnout‐related symptoms and acknowledging chronic sorrow. Furthermore, some parents also conveyed the desire to provide excellent care for their children, emphasizing that witnessing their child's positive qualities acted as a safeguard against burnout. Additionally, possessing self‐confidence and a positive outlook on life was deemed beneficial. Some parents emphasized the importance of adopting a positive mindset, finding meaning and joy in small things, approaching life 1 day at a time and letting go of worries, particularly those beyond one's control. This sentiment was echoed by P28, when asked what protected them from experiencing burnout:

I now live a bit from day to day. Enjoy each day. Today is today. And tomorrow is a new day, so to speak. I can put it very bluntly, don't worry about tomorrow's weather. That could change three more times, including at work. Of course, things have to happen at work and at home too. But we just try to enjoy the moment, and find joy in that and in your satisfaction. And yes, tomorrow is a new day, yes. It may sound very short‐sighted sometimes, but yes. Every day is a party, so to speak. [P28]

Moreover, not being overly concerned about others' opinions and practising self‐compassion was also considered helpful, as was adapting a practical approach by being solution‐oriented, organizing tasks and completing tasks.

**Setting priorities and boundaries, coming to terms with current circumstances and having physical complaints.** Although setting priorities and boundaries was considered beneficial, some parents found setting priorities and boundaries challenging due to the strong desire to do everything possible for their child. The third theme centred on accepting the current circumstances and choosing not to resist or struggle against the situation. This involved adjusting expectations and ambitions, for example in areas like work and career. Additionally, some parents identified past traumas—both related to the child and the parent's history—as a risk factor, constituting the fourth theme. Lastly, some parents also highlighted that experiencing physical complaints was a risk factor which was often linked to sleep deprivation from caring for the child with CCN.

#### 3.2.2 The Environment Interacting With the Parent

The category of environment interacting with the parent highlights the dynamic transactions between parents and other people and organizations. Six themes were found in this category, each outlined below.

**Organization of health and social care.** Some parents mentioned poor organization of health and social care (hereafter referred to as care) as a risk factor because of fragmentation, unsuitability, unavailability or the absence of care. The fragmentation manifested in the lack of coordination and continuity of care. Absence or unavailability of care was noted, for example, due to shortages of healthcare professionals or due to the scarcity of drivers for school transport. These organizational issues imposed a considerable burden on parents. Furthermore, care laws and regulations were noted for their lack of clarity, complexity, ongoing change and overall bureaucratic nature and therefore experienced as a risk factor. This bureaucratic nature forced parents to adhere to strict rules, set out to focus on process and protocol rather than flexibility to suit the child's and parents' needs, often resulting in excessive paperwork and meetings for parents. Some parents mentioned that they had to repeatedly demonstrate that their child needed care, creating the perception that prevailing laws and regulations prioritized mistrust and control rather than trust and support for the parent. Furthermore, some parents also pointed out that the CCN of the child did not fit into standardized laws, regulations and care provision, causing additional challenges for parents. The care provision for the child, along with the bureaucratic aspects, was considered a big burden; for example, one participant noted:

I know very well that having a care‐intensive child at home sooner or later I think you will end up there or at least go in that direction [refers to burnout]. […] The bureaucracy in the Netherlands is such that people spend half their time on a care‐intensive child and the other half of their time on the administration surrounding it. [P16]

In contrast, when care was considered well‐organized, it was a protective factor. When receiving suitable care and when understanding the care system and its prevailing laws and regulations, parents felt a sense of stability and resilience. This occurred alongside parents' confidence in and reliance on professionals, effective communication with them and assurance that care was well‐coordinated.

**Social support.** Parents mentioned the importance of receiving practical‐ and/or emotional support from friends, family, colleagues and professionals (e.g., from healthcare, municipality and schools). Some parents also mentioned that having a strong network and actively investing in it was helpful. Furthermore, for parents, support also entailed having access to psychological support or therapy for themselves. Conversely, the lack of acknowledgment from professionals regarding the challenges parents faced and the absence of recognition from municipalities for the care needed for the child was considered burdensome, as was having to independently assume the care provision for the child (e.g., when the partner was unable to help or when professional caregivers were sick or unavailable) or having no advocate or guiding support when navigating challenges. Due to the care complexities of the child, some parents mentioned that they did not have support in their closest network. When discussing how informal support protected from burnout, some parents expressed discomfort in delegating care responsibilities due to the complexities surrounding the child, as one parent explained:

We have a lot of help from my in‐laws. They are both retired. […] Yes and now it's better [points to the child] so I think soon we'll be able to deal with it more easily. But it was just, you have to imagine that he could walk to the window and then it could already be too much. Then he could have fallen over and be gone, so you don't want to do that to people, so to speak. But we do have a lot of nice people around us. [P15]

Asking, receiving and accepting help was acknowledged by some parents as an alleviating factor. Additionally, proactively seeking help for oneself and for the child, delegating and sharing caregiving responsibilities and organizing structured respite/live‐in care for the child with CCN was considered helpful.

**Conflicts.** Conflicts were mentioned to be related to work, family, (ex)partner and parenting‐related issues with (ex)partner. For example, the importance of avoiding conflict with the partner was noted:

I firmly believe that as partners you agree with each other on the big things […] because if you just don't agree with each other, that is not necessarily right or wrong. But if you end up in such a situation [refers to conflict with the other parent], I think that the risks you run of experiencing burnout are significantly higher. [P29]

**Self‐care.** Self‐care involved having the possibility and the (mental) space to take care of oneself. Attending to one's own personal needs was fundamentally important for sustaining care for the child and avoiding burnout. One parent advised other parents:

Well, take good care of yourself, make sure that you keep it up initially and in the end I think I have forgotten that and there is not enough attention for the fact that you as a parent also have to keep on going. I, at the end of the day, if you don't take care of yourself, you can't take care of your child. [P2]

Parents explained that it was helpful to devote attention to their physical, emotional and mental well‐being, encompassing activities that aligned with their desires and needs. Examples were seeking distractions, establishing a safe haven outside the home, engaging in energizing activities and making time for personal interests. Parents found self‐sacrifice, skipping rest and being overwhelmed by caregiving tasks unhelpful. Additionally, parents noted that multiple caregiving responsibilities and external concerns, such as family illness and death, hindered the possibility to engage in self‐care.

**Socio‐economic factors.** Some parents noted that experiencing financial concerns was a risk factor. Conversely, having the ability to balance work and personal life was seen as a protective. This included understanding and support from employers to work less or take time off. Furthermore, flexible work arrangements in terms of time, location and projects were noted to provide freedom. One parent recalled that having the choice to not work at all and thereby experiencing no associated work obligations prevented them from experiencing burnout:

I don't think I have any reason to suffer from a burnout. But what I really watch out for is work, so I don't work anymore and I would find it very intense if I still had to work as well, in addition to caring, because that would be too much, because caring for her and all the arranging it, so to speak, takes so much time. Uhm yes, I wouldn't want to work, but it's not that I don't want to do anything. I just can't handle the permanent job and the obligations that come with it. I can't deal with that. [P3]

Some parents, however, found that working and being able to go to work served as a refuge or respite from the continuous demands of caregiving and parenting at home.

**The nature and severity of the child's disability or disease.** This theme encompassed the complex, demanding and prolonged care requirements of the child, which at times involved recurring and medically complex tasks demanding continuous attention. Caring for the child often entailed ongoing attention, frequent hospital visits, numerous appointments and therapies causing practical challenges for the parents. On top of these challenges, the uncertainty of the prognosis, occasionally leading to acute and/or life‐threatening situations, was a source of stress and concern for the parents.

#### 3.2.3 The Sum of All Factors

In this category, four themes were identified, as described below.

**An accumulation of problems.** Parents underscored that burnout was not a result of a single factor but rather from a combination and interaction between various factors that hold significance for the individual parent. One parent described this in terms of important domains in life:

I do think that a combination of, umm (…) you have a number of domains within your life [refers to work, healthcare and relationship with partner]. If everything goes well and one domain doesn't go so well, you can usually deal with that. But if multiple problems arise in multiple domains, things go wrong. So if, in my case, you feel that you are failing at work and caring for your child, and you still have a lot to process and you also have no time for yourself, and you don't do fun things that give you energy and at a certain point you no longer get any energy from your work, then that is a recipe for stress. [P23]

**A risk factor is eliminated, the situation is stable/in order.** Some parents mentioned that the elimination of a risk factor was helpful in alleviating the challenges. Additionally, perceiving the situation as stable, namely, maintaining a balance between the available capacities/resources and challenges/demands was seen as beneficial.

**Having to endure.** Lastly, the theme *having to endure* revolved around the perception that caring for the child with CCN brought long‐term parental and caregiving responsibilities, leaving parents with no alternative but to persevere. Some parents viewed this as helpful, serving as an internal motivator to endure, while others perceived it as a burden with no end in sight. As such, the fourth theme constituted both a risk and a protective factor.

## 4 Discussion

The objective of this study was to investigate risk and protective factors according to the perspectives of parents of children with CCN. Fifteen distinct factors were found, comprising three main categories: ‘the parent’, ‘the environment interacting with the parent’ and ‘the sum of all factors’. While certain factors paralleled those observed in parents of children without CCN, others were distinctly tied to the unique demands of parenting and caregiving for a child highly dependent on the parent. Most factors were identified as both risk and protective factors, underscoring the dynamic nature of burnout.

Several themes identified in this study align with findings from research on burnout among parents in general, including conjugal disagreements, dispositional factors, social support, all of which have been well‐documented as risk factors for burnout (Le Vigouroux and Scola 2018; Mikolajczak, Raes, et al. 2018; Sorkkila and Aunola 2020, 2021). Considering the variations found in socio‐demographic factors from the literature, parents did not mention that factors such as their gender, age or marital status influenced burnout. However, work (e.g., work‐life balance, flexible work arrangements, an understanding employer) and financial strain (e.g., having the financial the possibility to not work) were considered important socio‐demographic factors, which is in line with other findings (e.g., Lindström et al. 2011). Importantly, the relevance of socio‐demographic characteristics of the parents does not lie in the variable itself, but rather in the consequences for parenting or caregiving tasks (Gérain and Zech 2019). For example, a poor financial situation may be due to limited employment opportunities related to the ongoing care for the child, and finding (ad hoc) practical support may be an additional challenge due to the complexity of caring for the child. Moreover, being a parent of a child with CCN appears to bring in a cascade of other risk factors peculiar to this context. For example, the nature and severity of the disability/disease cause challenges for the organization of health and social care, which evoke specific emotions within the parent (e.g., chronic sorrow). Given that this study only provides a broad overview of potential factors from the perspective of parents, a deeper comprehension of the dynamic development of burnout over time is needed. Therefore, future research should investigate the relative effects of these factors in larger, preferably cross‐national samples, ideally using a longitudinal design, and explore potential interrelationships between the factors.

One notable divergence from previous research was the limited emphasis parents placed on the parent–child relationship as a risk or protective factor for burnout. Although prior studies have found relationship quality to be associated with parental burnout (Gérain and Zech 2019; Ren et al. 2024), parents in this study did not explicitly identify it as such. A possible explanation is that having a child with CCN is perceived as a fixed reality, shifting parental focus towards the modifiable demands of caregiving. These care demands may overshadow the parent–child relationship in discussions about stress and resilience. Additionally, the interviews were framed around parents' perceptions of what contributes to or buffers against burnout in the context of raising a child with CCN, rather than focusing on how their emotional bond with the child might influence their experience of burnout. This could be a valuable area for further exploration in future research.

While burnout is commonly portrayed as an issue within the parent (Lindström et al. 2016), our study suggests that factors within the broader context in which parents are participating are just as crucial as those factors inherent to the parent. For example, issues surrounding the organization of care were mentioned as a risk factor by numerous participating parents, as well as support from formal and informal networks. Moreover, factors inherent to the parent, such as beliefs and attributions, are also the result of wider societal norms and values about parenting and caregiving. This underscores the importance of attention to the broader context of these parents, which extends beyond those factors inherent to the parent, encompassing also parents' social networks, the organization of health and social care along with the corresponding policies and legislations and even the prevailing values and norms in the wider society. As these factors could also work in a protective way, the community, society and the cultural context of parents may contribute to the development and to the prevention of burnout. There may be a need for an innovative, holistic and multidisciplinary approach that considers the wellbeing of the individual parent as an integral part of society, because efforts solely focusing on parents and professionals might not fully address the matter. As for current practice, this also means some care models will be more pertinent than others (i.e., integrated, federated, community‐based care).

Our findings suggest that burnout among parents of children with CCN is not caused by one single factor, but by the sum of all factors. Particularly unique to parenting a child with CCN is the obligation for parents to persevere beyond the normative tasks of parenting, as they cannot simply relinquish their responsibilities. As a result, burnout symptoms fluctuate, indicating that burnout is a dynamic state rather than a fixed trait or condition. This could be illustrated by a roly‐poly toy as a metaphor. The roly‐poly (parent) wobbles due to the internal weight in combination with environmental influences but always rights itself, the latter symbolizing the parental and caregiving responsibility, leaving no option but to persevere. In a positive light, the broad overview of risk and protective factors in this study provides a promising outlook. Diverse elements amendable to targeted support were found for alleviating and mitigating burnout, which not only offers avenues for intervention but also holds potential for developing preventive strategies aimed at reducing the risk of parental burnout. However, our model only provides a starting point to understand the complex mechanism behind the development of burnout. Future studies should look deeper into the specific mechanisms to understand the variations in and the specific weights of risk and protective factors, both at an individual and population‐wide level. The resource and demands model (Mikolajczak and Roskam 2018) could provide a viable framework for further exploring and adding risk and protective factors among parents of children with CCN.

## 5 Limitations

First, the participating parents were individuals with interest and ability to express their experiences with burnout. Furthermore, those parents who were deeply overwhelmed might have been less likely to participate, as participating in a study might not be a priority for them. Consequently, our findings are only indicative of a subset of parents. Nonetheless, during the recruitment of parents, attention was given to background variables and PBI scores, revealing variation in burnout‐related symptoms. Second, parents who did not identify or recognize the term ‘burnout’ were excluded. Some parents may not resonate with the term, viewing it as a non‐legitimate condition, or even a taboo (Mikolajczak et al. 2021). This does not negate the existence of similar thoughts and feelings among those who do not identify with the term. Future research should examine why some parents do not resonate with the term ‘burnout’ and explore potential differences in how they perceive and experience parenting a child with CCN. Such insights could help those parents who do resonate with the term. It is probable that similar factors, as found in this study, have an impact on parents' wellbeing beyond sole burnout. However, to gain a broad overview and to avoid a one‐sided perspective on risk and protective factors on burnout, we intentionally included parents who had not experienced burnout. Last, as this study was conducted among Dutch‐speaking parents living in the Netherlands, the risk and protective factors found in this study pertain to the cultural context, social expectations of parenting and attitudes toward children with CCN as these are confined in the parents' perceptions. Future research on risk and protective factors for burnout should be done across cultural contexts to further understand the role of cultural beliefs on parental burnout.

## 6 Conclusion

The results indicate that burnout might stem from an interplay of varied risk and protective factors that hold significance for individual parents, including context‐specific factors. These factors expand beyond those inherent to the parent, involving wider societal structures that stand incongruent with the way burnout is currently being approached. This indicates the need for an innovative, holistic and multidisciplinary approach personalized to the individual parent, which approaches wellbeing as an integral part of society. As parenting a child with CCN often is a long‐term task, leaving parents with no option but to persevere, it becomes crucial to consider which factors promote resilience and which risk factors can be removed or minimized. Findings suggest that multiple actors are needed to address burnout: parents themselves, the informal network, care professionals and the broader society. If everyone takes responsibility for those components which they can influence, then there is a promising outlook.

## Author Contributions

**Nathalie J. S. Patty:** conceptualization, data curation, formal analysis, project administration, supervision, validation, writing – original draft, writing – review and editing, funding acquisition, methodology, investigation. **Karen M. van Meeteren:** investigation, conceptualization, formal analysis, funding acquisition, methodology, project administration, supervision, validation, writing – original draft, writing – review and editing. **Minke Verdonk:** conceptualization, formal analysis, funding acquisition, investigation, methodology, project administration, writing – review and editing. **Marjolijn Ketelaar:** conceptualization, formal analysis, funding acquisition, supervision, methodology, validation, writing – review and editing. **Carlo Schuengel:** writing – review and editing, conceptualization, formal analysis, funding acquisition, methodology, supervision, validation. **Agnes M. Willemen:** conceptualization, funding acquisition, formal analysis, methodology, supervision, validation, writing – original draft, writing – review and editing.

## Ethics Statement

Ethical approval was obtained from the Scientific and Ethical Review Board of the Faculty of Behavioural and Movement Sciences of the Vrije Universiteit Amsterdam (registration numbers: VCWE‐2020‐147 and VCWE‐2021‐078).

## Consent

Informed consent was obtained from all participants.

## Conflicts of Interest

The authors declare no conflicts of interest.

## Supporting information


**Data S1.** Supporting information.

## Data Availability

The data that support the findings of this study are available upon reasonable request.

## Endnotes

^1^
MBI‐Human Services Survey—MBI‐HSS: Copyright 1981 Christina Maslach & Susan E. Jackson. All rights reserved in all media. Published by Mind Garden Inc., www.mindgarden.com.
